# Epidemiology and impact of hallux valgus: more than just bunions

**DOI:** 10.1186/1757-1146-4-S1-A8

**Published:** 2011-05-20

**Authors:** Edward Roddy

**Affiliations:** 1 Arthritis Research UK Primary Care Centre, Keele University, Staffordshire, ST5 5BG, UK

## 

Hallux valgus (HV) is a common deformity characterized by progressive lateral deviation of the great toe at the first metatarsophalangeal joint (1^st^ MTPJ). It commonly associates with a painful overlying soft-tissue prominence, the “bunion”. A recent systematic review estimated the prevalence of HV to be 23% in adults aged 18 to 65 years. It becomes more frequent with increasing age and is more prevalent in women than men. Several potential risk factors for HV have been identified. Various mechanical factors are thought to associate with HV including metatarsal length and head-shape, first ray hypermobility and foot posture. HV is rare in unshod populations but associates with wearing high-heeled or narrow shoes. The relationship between HV and obesity is less clear. Some studies have found an association between HV and increasing body mass index (BMI) whereas others have found no association. Most recently, it has been suggested that the association between HV and BMI differs between the genders, with a lower prevalence with increasing BMI in women but no association in men.

HV poses a significant health problem, and associates with foot pain, poor balance, gait impairment, immobility, and risk of falling. The likelihood of 1^st^ MTPJ osteoarthritis (OA) increases with HV severity but nodal OA and pain at the low back, hip and knee are also associated with HV, suggesting that it is a component of generalised OA. Several recent studies have examined the relationship between HV and health-related quality of life (HRQOL). Symptomatic HV appears to associate with reduced HRQOL. However, both general and foot-specific HRQOL are progressively lower with increasing severity of HV deformity, regardless of foot pain. Importantly, the association of both the presence and severity of HV with impaired HRQOL is not limited to pain and physical function but extends to general health, vitality, social function, and mental health. In summary, the impact of HV extends beyond local influence on foot OA, balance, gait and falls, to impair HRQOL. Future prospective studies are required to identify risk factors for the development and progression of HV and hence possible targets for prevention and intervention.

